# Microbiota of the Small Intestine Is Selectively Engulfed by Phagocytes of the Lamina Propria and Peyer’s Patches

**DOI:** 10.1371/journal.pone.0163607

**Published:** 2016-10-04

**Authors:** Masatoshi Morikawa, Satoshi Tsujibe, Junko Kiyoshima-Shibata, Yohei Watanabe, Noriko Kato-Nagaoka, Kan Shida, Satoshi Matsumoto

**Affiliations:** Yakult Central Institute, Tokyo, Japan; New York State Department of Health, UNITED STATES

## Abstract

Phagocytes such as dendritic cells and macrophages, which are distributed in the small intestinal mucosa, play a crucial role in maintaining mucosal homeostasis by sampling the luminal gut microbiota. However, there is limited information regarding microbial uptake in a steady state. We investigated the composition of murine gut microbiota that is engulfed by phagocytes of specific subsets in the small intestinal lamina propria (SILP) and Peyer’s patches (PP). Analysis of bacterial 16S rRNA gene amplicon sequences revealed that: 1) all the phagocyte subsets in the SILP primarily engulfed *Lactobacillus* (the most abundant microbe in the small intestine), whereas CD11b^hi^ and CD11b^hi^CD11c^hi^ cell subsets in PP mostly engulfed segmented filamentous bacteria (indigenous bacteria in rodents that are reported to adhere to intestinal epithelial cells); and 2) among the *Lactobacillus* species engulfed by the SILP cell subsets, *L*. *murinus* was engulfed more frequently than *L*. *taiwanensis*, although both these *Lactobacillus* species were abundant in the small intestine under physiological conditions. These results suggest that small intestinal microbiota is selectively engulfed by phagocytes that localize in the adjacent intestinal mucosa in a steady state. These observations may provide insight into the crucial role of phagocytes in immune surveillance of the small intestinal mucosa.

## Introduction

Abundant phagocytes, such as dendritic cells (DCs) and macrophages, are distributed in the small intestinal mucosa [1–3]. In contrast to the large intestine, which is heavily colonized by gut bacteria, the small intestinal mucosa is frequently exposed to various exogenous antigens, such as food ingredients and microbial components. In addition, substantial numbers of gut microbes have been reported to colonize the murine small intestine [4]. The small intestinal mucosa is equipped with an immune surveillance system that maintains homeostasis. The continuous sampling of the microbiota by mucosal phagocytes contributes to the immune balance.

Several pathways for microbial uptake by phagocytes have been reported. For example, M cells distributed throughout the follicle-associated epithelium in Peyer’s patches (PP) are believed to transcytose luminal antigens in a selective manner, resulting in the subsequent uptake of these antigens by phagocytes residing in the subepithelial dome [5]. Moreover, some types of phagocytes sample luminal antigens by extending their dendrites between enterocytes or through M cells, although this has not yet been observed under physiological conditions [6, 7].

It has become clear that uptake of microbial antigens can shape host immunity. Antigens of segmented filamentous bacteria (SFB), which has been shown to colonize murine small intestines and trigger various host adaptive immune responses such as IgA production and Th17 cell expansion [4, 8], are presented by small intestinal antigen-presenting cells and drive antigen-specific Th17 cell differentiation, according to a recent report [9]. Moreover, a CD4CD8αα T cell subset identified as Foxp3^−^ IL-10-secreting regulatory T cells exhibited a repertoire that was highly skewed toward the recognition of *Faecalibacterium prausnitzii*, a dominant *Clostridium* species in the healthy human gut microbiota [10].

Considering that the small intestinal mucosa remains in a homeostatic state although it is frequently exposed to various antigens, small intestinal phagocytes are thought to play a crucial role in maintaining mucosal homeostasis by sampling the luminal gut microbiota. However, there is little information on microbial uptake by these cells and the effects of this uptake on mucosal immunity in a steady state. Here, we report on our study to elucidate microbial uptake by phagocytes of specific subsets in the small intestinal lamina propria (SILP) and PP of specific-pathogen-free (SPF) C57BL/6 mice. We also investigated the composition of the gut microbiota engulfed by phagocytes of these subsets and analyzed cytokine gene expression from these cells, as well as their abilities to induce helper T cell subsets from naïve T cells.

## Materials and Methods

### Mice

C57BL/6 female mice, 8 weeks of age, were obtained from CLEA Japan and were maintained for 1–2 weeks under SPF conditions at Yakult Central Institute, prior to use in this study. B6.Cg-Tg(TcraTcrb)425Cbn/J (OT-II) mice were obtained from The Jackson Laboratory and maintained in the animal facility at the Institute. OT-II female mice, 9–15 weeks of age, were used for this study. Mice were sacrificed by exsanguination after isoflurane inhalation, prior to sampling the small intestine. All of the animal experiments in this study were approved by the Institutional Animal Care and Use Committee of Yakult Central Institute (Permit Number: 13–0346, 14–0022, 14–0053, 14–0163, and 14–0210).

### Cells and small intestinal contents

SILP cells were prepared according to a method described in a previous report [11], with some modifications. The small intestine from the pyloric sphincter to the ileocecal junction was excised and rinsed in ice-cold phosphate-buffered saline (PBS). After removal of the mesentery and PP, the intestine was opened longitudinally, washed of its contents with ice-cold PBS, and cut into 1-cm pieces. The intestinal segments were treated twice with Hank’s balanced salt solution (HBSS, Life Technologies) containing 0.45 mM DL-Dithiothreitol (Sigma), and then incubated with HBSS containing 0.45 mM DL-Dithiothreitol and 2 mM EDTA for 20 min each at 37°C with agitation. After removal of the epithelial layer by decantation, the resulting intestinal segments were rinsed with RPMI 1640 (Wako) containing 10 mM HEPES, a 0.5% penicillin-streptomycin antibiotic mixture (Ab mix, Sigma), and 5% fetal bovine serum (FBS, SAFC Biosciences), and incubated with RPMI 1640 medium containing 10 mM HEPES, 0.5% Ab mix, 10% FBS, 4 U/ml Collagenase-Yakult (Yakult Pharmaceutical), and 2.5 μg/ml DNase I (Sigma) twice for 1 hour at 37°C, with gentle agitation in a CO_2_ incubator. The supernatants were pooled and pelleted by centrifugation. The obtained cell suspension was termed SILP cells. PP cells were prepared according to the above protocol for SILP cell preparation. In brief, PP excised from small intestines were washed in HBSS containing 0.5% Ab mix and then incubated with RPMI 1640 containing 10 mM HEPES, 0.5% Ab mix, 10% FBS, 80 U/ml Collagenase-Yakult, and 50 μg/ml DNase I twice for 1 hour at 37°C with gentle agitation in a CO_2_ incubator. The obtained cell suspension was termed PP cells. The small intestinal content was obtained by pelleting the wash solution from the longitudinally opened small intestines by centrifugation at 8370 × *g* for 15 min and then resuspension of the pellet in ice-cold PBS.

### Flow cytometry

SILP and PP cells were incubated with FITC-conjugated anti-CD11b (clone M1/70) and PE-Cy7-conjugated anti-CD11c (clone N418), and phagocyte subsets were sorted on the basis of their CD11b and CD11c expression, using a MoFlo^TM^ XDP (Beckman Coulter). The sorted cells were collected in ice-cold FBS during the sorting operation to maintain survival rates. The cell surface expression pattern in each subset was examined using Allophycocyanin (APC)–conjugated anti-CD103 (clone 2E7), APC-conjugated anti-I-A/I-E (major histocompatibility complex class II; MHC-II, clone M5/114.15.2), APC-conjugated anti-CD64 (clone X54-5/7.1), PE-conjugated anti-CD24 (clone M1/69), PE-conjugated anti-CX3CR1 (clone SA011F11), PE-conjugated anti-F4/80 (clone BM8), PE-conjugated anti-Siglec-F (clone E50-2440, BD Pharmingen), biotin-conjugated anti-CD8α (clone 53–6.7), and PE-conjugated streptavidin after FcR blocking with anti-CD16/32 (clone 93). Dead cells were eliminated using a Zombie Aqua^TM^ Viability Kit. All reagents were purchased from BioLegend unless otherwise noted.

### Microscopic observation

The sorted cells were stained with Giemsa stain or subjected to fluorescence in situ hybridization (FISH) with a Eub338 probe targeting a conserved region in the bacterial 16S rRNA. Sorted cell specimens were prepared by Cytospin 3 centrifugation (Shandon), then fixed with 3% paraformaldehyde overnight at 4°C, and permeabilized with 96% ethanol for 10 min at room temperature. For the FISH analysis, specimens were incubated with a hybridization solution (750 mM NaCl, 100 mM Tris-HCl, 5 mM EDTA, 0.01% BSA, and 10% dextran sulfate) containing 4.5 ng/μl of Cy3-conjugated Eub338 probe (GCTGCCTCCCGTAGGAGT), overnight at 40°C [12]. After incubation with a wash solution (50 mM NaCl, 4 mM Tris-HCl, and 0.02 mM EDTA) for 20 min at 45°C and air drying, the specimens were stained with TO-PRO^®^-3 (Life Technologies) and embedded in VECTASHIELD^®^ (Vector Laboratories). The Giemsa-stained specimens were observed with a BX51 light microscope (Olympus), and the FISH specimens were observed with an LSM510 (Carl Zeiss) or a TCS SP8 (Leica) confocal laser scanning microscope.

### Cytokine gene expression levels

To characterize the immune properties of each phagocyte subset, we analyzed the cytokine gene expression levels in these cells by quantitative reverse transcription PCR. The total RNA of each subset was extracted as described above and reverse transcribed using a High-Capacity cDNA Reverse Transcription Kit. Gene expression levels in the resulting cDNA were analyzed using TaqMan^®^ Universal PCR Master Mix and TaqMan^®^ Gene Expression Assays for *Il12b*, *Il10*, *Tgfb1*, *Tnfa*, *Il6*, and *Il23a* on an Applied Biosystems^®^ 7500 Real Time PCR System. *Gapdh* was used as an endogenous control to normalize gene expression levels. All reagents were purchased from Applied Biosystems.

### Helper T cell differentiation-inducing properties

To further characterize phagocyte subsets, we evaluated their abilities to induce helper T cell subsets from naïve T cells in the steady state. Each of the phagocyte subsets (1 × 10^4^ cells) was cocultured with OT-II splenic CD62L^+^CD44^-^CD4^+^ T cells (3 × 10^5^ cells) and albumin from chicken egg white (0.3 mg/ml, Sigma) in 200 μl of RPMI 1640 medium containing 0.1% 2-mercaptoethanol, 0.5% AB mix, and 10% FBS in a 96-well flat-bottom plate. After 3.5 days, cells were harvested and restimulated with Leukocyte Activation Cocktail with BD GolgiPlug^TM^ (BD Pharmingen) for 5 hours. The resulting cells were stained with PE-conjugated anti-CD4 (clone RM4-5) after FcR blocking with anti-CD16/32, permeabilized using BD Cytofix/Cytoperm^TM^ (BD Pharmingen), and incubated with several sets of anti-cytokine monoclonal antibodies as described below. Percentages of cytokine-positive cells among CD4^+^ T cells were measured in a Gallios^TM^ Flow Cytometer (Beckman Coulter). OT-II splenic naïve T cells were stained with PE-Cy7-conjugated anti-CD62L (clone MEL-14), PE-conjugated anti-CD44 (clone IM7), and FITC-conjugated anti-CD4 (clone RM4-5), while intracellular cytokines were stained with Alexa Fluor 647 (AF647)-conjugated anti-IFN-γ (clone XMG1.2), AF647-conjugated anti-IL-17 (clone TC11-18H10.1), Brilliant Violet 421 (BV421)-conjugated anti-IL-4 (clone 11B11), and BV421-conjugated anti-IL-10 (clone JES5-16E3). All reagents were purchased from BioLegend unless otherwise noted.

### Nucleic acid extraction

Total DNA and RNA from the sorted cells was extracted using an AllPrep DNA/RNA Micro Kit (Qiagen) after washing the cells twice with HBSS containing 0.5% Ab mix. Total DNA from the small intestinal content was extracted using the bead-phenol method as described previously [13]. In brief, a suspension of the small intestinal content was agitated vigorously for 30 sec using a FastPrep^®^ FP120 Cell Disrupter (Thermo Savant) at a power level of 5.0, after supplementation with 0.3 g of glass beads (diameter: 0.1 mm, TOMY), 500 μl of TE-saturated phenol, 50 μl of 10% SDS, and 250 μl of extraction buffer (200 mM Tris-HCL and 80 mM EDTA; pH9.0). After centrifugation at 20630 × *g* for 5 min, the supernatant was collected. The small intestinal DNA was obtained by subsequent phenol-chloroform extractions and an isopropanol precipitation.

### Bacterial 16S rRNA gene amplicon sequencing

To elucidate the composition of the gut microbiota that was engulfed by the phagocyte subsets, bacterial 16S rRNA gene amplicon sequencing was performed. A V4 region of the bacterial 16S rRNA gene was amplified from the DNA of phagocytes of various subsets and from the small intestinal contents using primer set F515 and R806 as described previously [14], with some modifications. PCR reactions were prepared with 25 μl of SYBR^®^ Premix Ex Taq^TM^ II (Takara Bio), 1 μl of each of the primers, 5 μl of the extracted DNA, and 18 μl of RNase-free water (Ambion). PCR conditions included an initial step at 50°C for 2 min and at 95°C for 10 min, with a subsequent amplification step at 95°C for 30 sec, at 55°C for 30 sec, and at 72°C for 90 sec for repeated cycles on an Applied Biosystems^®^ 7500 Real Time PCR System (Applied Biosystems). The amplification step was stopped before the fluorescent intensity reached a plateau. The resulting amplicons were purified using Agencourt^®^ AMPure^®^ XP (Beckman Coulter), quantified using a Quant-iT^TM^ PicoGreen^®^ dsDNA Assay Kit (Life Technologies), pooled in equimolar amounts, and then sequenced on an Illumina MiSeq platform, using a MiSeq Reagent Kit v2 (Illumina) as described previously [15].

### Bioinformatics analysis

The 16S rRNA gene sequences were analyzed using the Quantitative Insights Into Microbial Ecology (QIIME) software package version 1.8.0 as described previously [16]. In brief, raw 250-bp paired-end sequence reads were combined using the fastq-join script [17], with the minimum allowed overlap in base pairs required to join pairs set at 150 bp and the maximum allowed percentage difference within a region of overlap set at 15%. Quality filtering was performed as described previously [18], except with parameters q = 24 and c = 0.0005%. Further data processing comprised clustering of sequences with less than 3% dissimilarity using the USEARCH algorithm version 5.2.32 [19] with an open-reference operational taxonomic unit (OTU) clustering method in the Greengenes database (13_8; greengenes.lbl.gov/), and detecting and removing chimeras using the UCHIME algorithm [20]. The most abundant sequence in each OTU was selected as the representative sequence, and the resulting OTUs were assigned to taxa using the Ribosomal Database Project classifier [21], trained by the Greengenes reference database [22] via QIIME and set at a minimum confidence score of 80%. The sequences of OTUs were aligned against the Greengenes core reference alignment using the Python Nearest Alignment Space Termination alignment algorithm in QIIME [23]. The most closely related known species to each OTU was determined by means of a local BLAST search of the All-Species Living Tree Project database [24], except that OTU ID #1058952 was identified as *Candidatus Arthromitus* (SFB) using a BLAST search of the National Center for Biotechnology Information database (http://www.ncbi.nlm.nih.gov/). Sequence data were deposited in the DNA Data Bank of Japan Sequence Read Archive under BioProject Accession No. PRJDB4392 (http://www.ddbj.nig.ac.jp/).

### Statistical analysis

The statistical significance of differences in the means ± standard deviations among the phagocyte subsets were calculated using nonparametric Tukey test for multiple comparisons. *P* values of less than 0.05 were considered statistically significant.

## Results

### Isolation of phagocyte subsets

**Fig 1:**
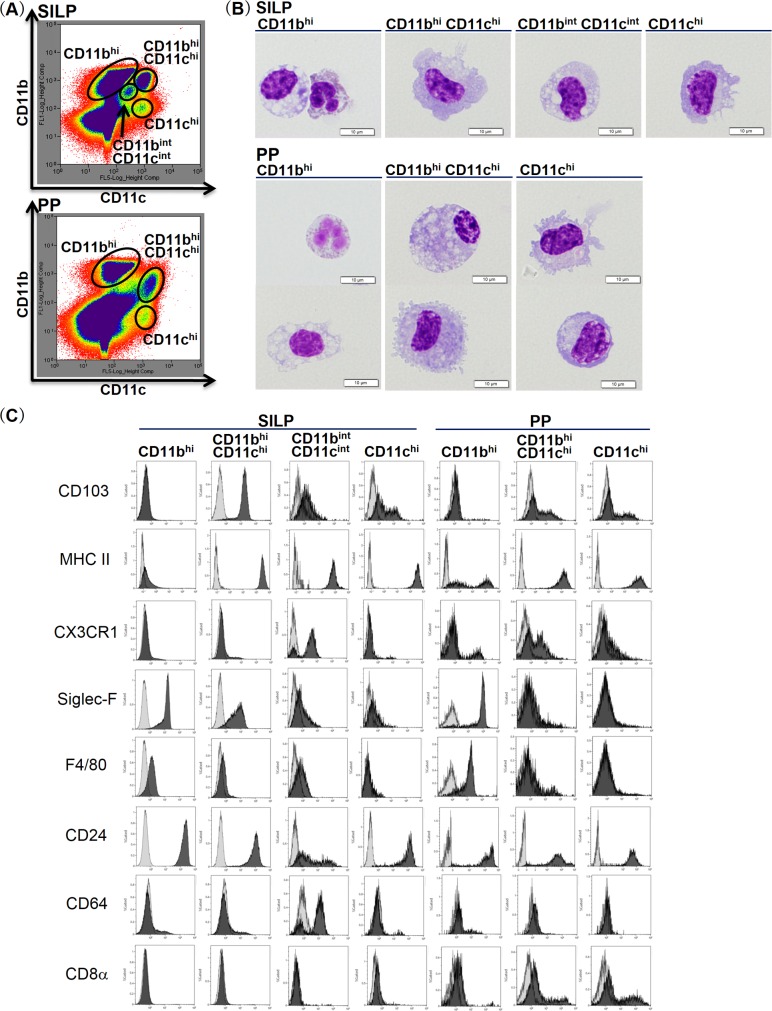
Isolation of phagocyte subsets from the SILP and PP in the steady state. (A) SILP and PP cells were prepared from the small intestines of C57BL/6 mice and were divided into 4 or 3 subsets, respectively, on the basis of CD11b/CD11c expression patterns determined by cell sorting. (B) Each subset was stained with Giemsa stain and observed under a light microscope at a magnification of 1000. Scale bars represent 10 μm. (C) The cell surface expression pattern of the indicated antigens in each subset was examined using a cell analyzer. Histograms were pregated on live cells and then into the indicated subsets as defined above. Light gray histograms represent isotype controls.

On the basis of CD11b/CD11c expression patterns, SILP cells were divided into four subsets, CD11b^hi^ cells, CD11b^hi^CD11c^hi^ cells, CD11b^int^CD11c^int^ cells, and CD11c^hi^ cells, as reported previously (Fig 1A) [1]. CD11b^hi^CD11c^hi^ cells and CD11c^hi^ cells were morphologically homogeneous populations with single nuclei and dendrites, while CD11b^int^CD11c^int^ cells consisted of macrophage-like cells with single nuclei and vacuoles (Fig 1B). These mononuclear phagocytes, which exhibited an MHC-II^+^ phenotype, have been reported as distinct cell subsets distinguished by the expression patterns of CD64, CD24, CD103 and CX3CR1 [3]. CD11b^hi^CD11c^hi^ cells and CD11c^hi^ cells showed high levels of CD103 and CD24 expression (Fig 1C), indicating that these cell subsets are equivalent to CD103^+^CD11b^+^ DCs or CD103^+^CD11b^-^ DCs, respectively [3]. CD11b^int^CD11c^int^ cells contained an MHC-II^+^CD64^+^CX3CR1^+^ population of tissue-resident macrophages (Fig 1C) [3]. The other population, CD11b^hi^ cells of the SILP, primarily comprised eosinophils with segmented nuclei and acidophilic granules, and a minority of macrophage-like cells that exhibited the same morphological properties as those described above (Fig 1B). Previous studies have identified MHC-II^-^Siglec-F^+^ eosinophils and MHC-II^+^Siglec-F^-^ macrophages, both of which displayed F4/80^+^ phenotypes, in the CD11b^+^CD11c^-^ cell fraction of the murine SILP [25]. In this study, CD11b^hi^ cells appeared to be a homogenous population with an MHC-II^-^CD24^hi^F4/80^lo^Siglec-F^hi^ phenotype, indicating that most of the cell subset consists of eosinophils.

PP cells were divided into three subsets, CD11b^hi^ cells, CD11b^hi^CD11c^hi^ cells, and CD11c^hi^ cells (Fig 1A). In contrast to the SILP cell subsets, each PP cell subset was represented by a morphologically and phenotypically heterogeneous population (Fig 1B and 1C). Previous studies have identified CD11b^+^CD8α^-^ myeloid DCs, CD11b^-^CD8α^+^ lymphoid DCs, and CD11b^-^CD8α^-^ DCs in the MHC-II^hi^CD11c^+^ cell fraction of murine PP [26]. In this study, CD11b^hi^CD11c^hi^ cells and CD11c^hi^ cells of PP consisted of DCs and macrophage-like cells that exhibited the same morphological properties as those described above, both of which showed a high level of MHC-II expression and contained CD8α^+^ populations (Fig 1B and 1C). According to the previous studies, the PP CD11b^hi^CD11c^hi^ cell subset appeared to contain CD11b^+^CD8α^-^ myeloid DCs, while the PP CD11c^hi^ cell subset appeared to contain both CD11b^-^CD8α^+^ lymphoid DCs and CD11b^-^CD8α^-^ DCs [26]. In contrast, CD11b^hi^ cells of PP displayed a CD8α^-^F4/80^mid^Siglec-F^hi^ phenotype and were identified as eosinophils and macrophage-like cells based on their morphological properties, which likely corresponded to the MHC-II^+^ or MHC-II^-^ population, respectively.

### Each phagocyte subset showed distinct immune properties

**Fig 2:**
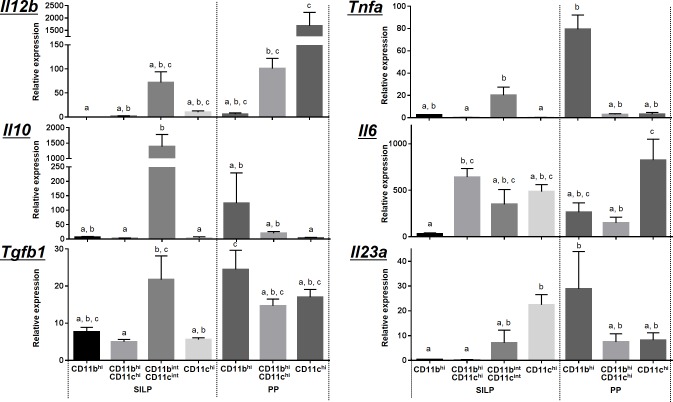
Cytokine gene expression levels of the phagocyte subsets. Total RNA was extracted from each phagocyte subset from the SILP and PP, and the gene expression levels of IL-12p40, IL-10, TGF-β, TNF-α, IL-6 and IL-23p19, which are cytokines secreted by DCs and macrophages, were analyzed using quantitative reverse transcription PCR. *Gapdh* was used as an endogenous control to normalize the levels of gene expression. Data represent the means and standard deviations of four independent experiments. Seven or eight C57BL/6 mice were pooled and used for PP or SILP experiments, respectively. Statistical analysis was performed using nonparametric Tukey test for multiple comparisons. There are significant differences between bars not sharing a common letter. *P* values of less than 0.05 were considered statistically significant.

In order to characterize the phagocyte subsets in the steady state, we analyzed the gene expression levels of IL-12p40, IL-10, TGF-β, TNF-α, IL-6 and IL-23p19, which are cytokines secreted by DCs and macrophages (Fig 2). High levels of *Il12b* expression were observed in the SILP CD11b^int^CD11c^int^ cell subset and in PP CD11b^hi^CD11c^hi^ and CD11c^hi^ cell subsets. In contrast, *Il10* expression levels were the highest in CD11b^int^CD11c^int^ cell subset from the SILP and CD11b^hi^ cell subset from PP. *Tnfa* expression was characteristic of CD11b^int^CD11c^int^ cell subset from the SILP and CD11b^hi^ cell subset from PP, both of which included macrophage-like cells.

**Fig 3:**
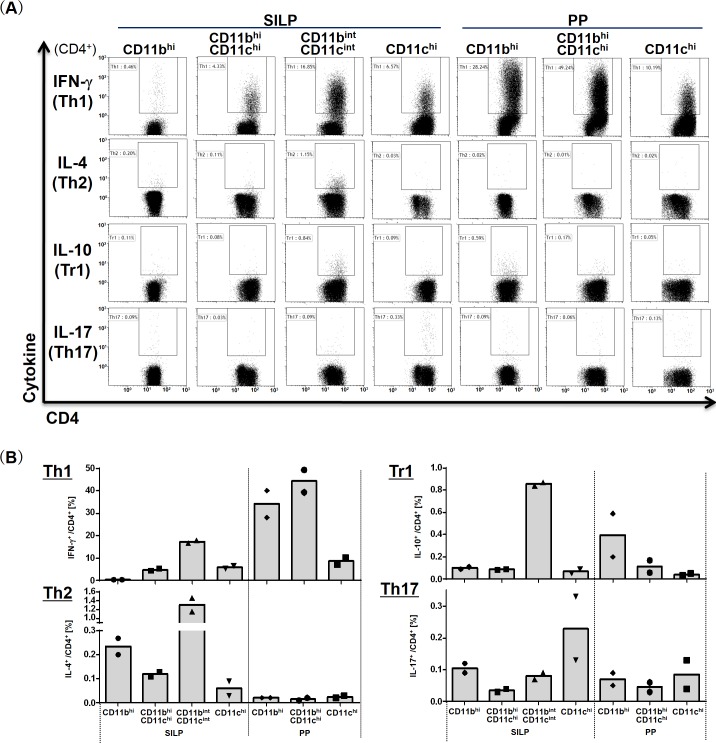
Helper T-cell differentiation-inducing properties of the phagocyte subsets. Each of the phagocyte subsets (1 × 10^4^ cells) was cocultured with OT-II splenic CD62L^+^CD44^-^CD4^+^ T cells (3 × 10^5^ cells) in the presence of albumin from chicken egg white for 3.5 days and then stained intracellularly for the indicated cytokines after restimulation for 5 hours with Leukocyte Activation Cocktail with BD GolgiPlug^TM^. Percentages of the indicated cytokine-positive cells among CD4^+^ T cells were determined by flow cytometry (A) and plotted with dots in bar graphs (B). Bars represent means of two independent experiments. Seven or eight C57BL/6 mice were pooled and used for each experiment.

In addition, we evaluated the abilities of these cell subsets to induce conversion of naïve T cells to helper T cells (Fig 3). Consistent with the results of gene expression analysis, CD11b^int^CD11c^int^ cell subset from the SILP and CD11b^hi^ and CD11b^hi^CD11c^hi^ cell subsets from PP, all of which showed high levels of *Il12b* or *Il23a* expression, were able to induce IFN-**γ**-producing CD4^+^ T (Th1) cells. Cells in CD11b^int^CD11c^int^ subset from the SILP, which expressed high levels of *Il10*, were found to be potent inducers of IL-4-producing CD4^+^ T (Th2) cells and IL-10-producing CD4^+^ T (Tr1) cells. Previous studies showed that the SILP CD103^+^CD11b^+^ DCs, equivalent to CD11b^hi^CD11c^hi^ cells of the SILP in this study, could induce IL-17-producing CD4^+^ T (Th17) cells [1, 2]. However, the percentage of Th17 cells induced by the cell subset was 0.04%, while that of Th17 cells induced by CD11c^hi^ cell subset of the SILP was 0.23%. The difference was slight but appreciable, suggesting that the SILP CD11c^hi^ cells, not CD11b^hi^CD11c^hi^ cells, were the most potent inducers of Th17 cells, which is consistent with the high levels of both *Il6* and *Il23a* expression. Another study demonstrated that the SILP CD103^+^CD11b^+^ DCs expressed high levels of IL-23p19 and IL-12p40 after bacterial flagellin administration [27]. Because mice maintained under SPF conditions were used in this study, the lack of bacteria possessing flagellin may lead to lower induction of Th17 cells by the SILP CD11b^hi^CD11c^hi^ cells, indicating that the ability to induce helper T cell subsets varies based on the microbial profile. Taken together, these data reveal the immune functions of the phagocyte subsets in the steady state and confirm that each subset exhibited distinct immune properties.

### Each phagocyte subset engulfed gut microbiota in the steady state

**Fig 4:**
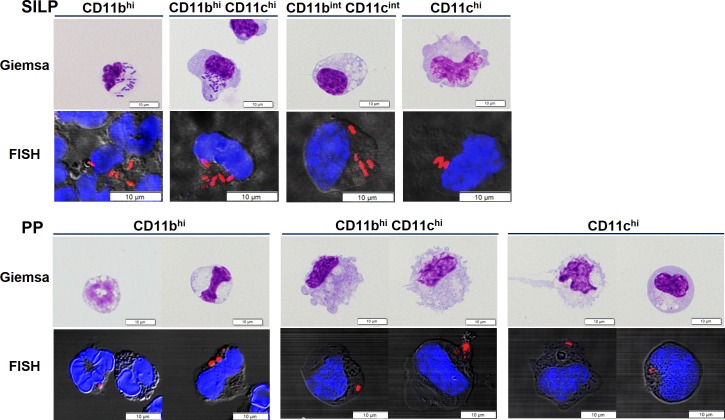
Microscopic observations of gut microbiota engulfed by phagocytes of specific subsets. Microscopic observation of each subset was performed at a magnification of 1000 or 630 after Giemsa staining or FISH with a Cy3-conjugated Eub338 probe (total bacteria, red) and TO-PRO^®^-3 (nuclei, blue), respectively. Scale bars represent 10 μm.

Among the Giemsa-stained cells, a small population of the SILP CD11b^hi^ and CD11b^hi^CD11c^hi^ phagocytes that had engulfed bacteria were observed under a light microscope (Fig 4). In order to determine whether microbes were engulfed by cells of other subsets, we hybridized bacterial 16S rRNA with a Eub338 probe (Fig 4). Phagocytes harboring bacterial 16S rRNA hybridized with the Eub338 probe were observed not only in both cell subsets described above, but also in the other cell subsets from the SILP and PP. These observations suggest that gut microbiota is engulfed by small intestinal phagocytes even in the steady state.

### Indigenous gut bacteria were selectively engulfed by phagocytes of specific subsets

**Fig 5:**
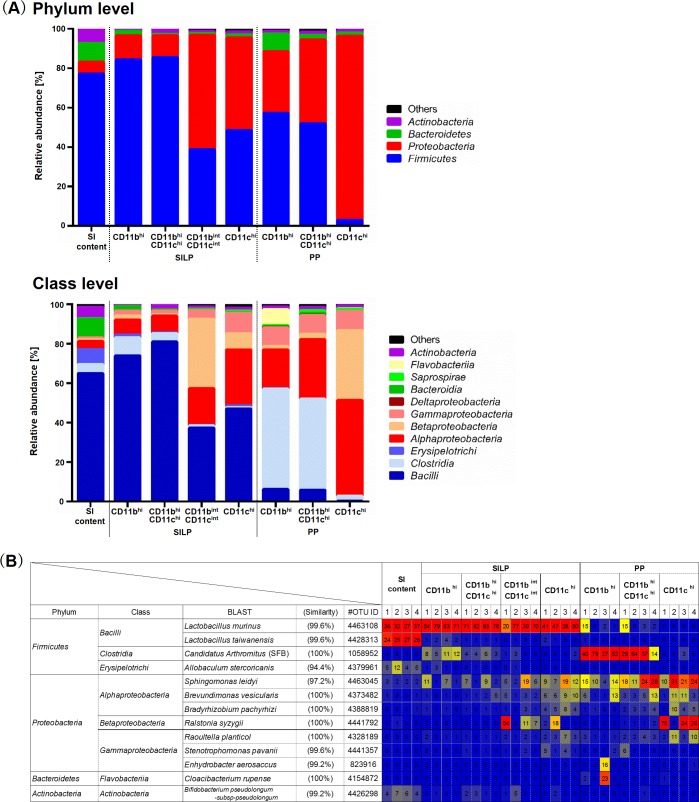
Composition of gut microbiota engulfed by phagocytes of specific subsets. Total DNA from each phagocyte subset and small intestinal content (SI content) was extracted and used for bacterial 16S rRNA gene amplicon sequencing. (A) The microbial composition of each sample at the phylum or class level. Data represent means of four independent experiments. (B) Heat map of each sample at the OTU level. Values represent the microbial composition (%) in each experiment (1–4). Seven or eight C57BL/6 mice were pooled and used for PP or SILP experiments, respectively.

Next, we examined the composition of gut microbiota engulfed by the phagocytes using bacterial 16S rRNA gene amplicon sequencing (Fig 5). First, the sequences demonstrated that the composition of microbes detected in all subsets was different from that of the small intestinal contents (Fig 5A). The microbial composition in phagocytes was dominated mostly by *Firmicutes* and *Proteobacteria*. Compared to the small intestinal contents, every subset had a lower percentage of *Actinobacteria* and *Bacteroidetes*. Second, the heat map of OTUs showed that cells of all subsets from the SILP engulfed more *Lactobacillus*, the most abundant microbial population in the small intestinal lumen, than cells of subsets from PP, whereas CD11b^hi^ and CD11b^hi^CD11c^hi^ cell subsets from PP engulfed more *Candidatus Arthromitus* (SFB), an indigenous bacterium reported to adhere to intestinal epithelial cells (Fig 5B) [4]. Most *Proteobacteria* detected in all subsets were also found in the negative control, an FBS fraction prepared in the sorting step and subsequently subjected to DNA extraction and PCR steps, similar to the cell subset fractions (data not shown), raising the possibility that most of the *Proteobacteria* detected in this study was derived from the reagents used in the several steps described above. It is not clear whether the *Proteobacteria* were engulfed by the phagocytes in this study, although *Proteobacteria* in PP have been reported [28]. Further investigations, such as isolation of the bacteria in each cell subset, will be required to clarify this. Third, the heat map also suggests that, among the *Lactobacillus* species engulfed by the SILP cell subsets, *L*. *murinus* was engulfed much more frequently than *L*. *taiwanensis*, even though both of these *Lactobacillus* species were abundant in the small intestinal lumen (Fig 5B). These results suggest that gut bacteria such as *L*. *murinus* and SFB are selectively taken up by phagocytes in the steady state.

## Discussion

This study, using SPF C57BL/6 mice under physiological conditions, revealed that *Lactobacillus* were engulfed frequently by all of the phagocyte subsets from the SILP, whereas SFB were engulfed frequently by CD11b^hi^ and CD11b^hi^CD11c^hi^ cell subsets from PP (Fig 5B). In addition, *L*. *murinus* was engulfed much more frequently than *L*. *taiwanensis* by the SILP subsets, while both *Lactobacillus* species were abundant in the small intestinal lumen (Fig 5B). *L*. *murinus* and SFB are indigenous bacteria that colonize the murine small intestine [4]. It is interesting that these bacteria were selectively engulfed by phagocytes in the steady state. This new finding prompts at least two questions.

First, what determines the selectivity of microbial uptake? The data in this study suggest that the accessibility to luminal microbiota differs among phagocytes of the various subsets, and that the susceptibility to uptake by phagocytes is different among indigenous gut bacteria. These differences may have two possible explanations. The first possibility is that there are differences in the distribution and localization of phagocytes and luminal bacteria. The distribution of phagocyte subsets has been reported to differ in the duodenum, jejunum, and ileum [2]. Similarly, the distribution of luminal gut bacteria is thought to vary in the small intestine along a longitudinal axis. These differences in distribution might result in the differences in accessibility to certain indigenous bacteria. In addition, a bacterium considered to be *L*. *murinus* has been observed on the surface of colonic epithelial cells in mice refed after starved for 36 hours [29], and SFB have been found to adhere to small intestinal epithelial cells [4]. These differences in luminal localizations of bacteria in the intestine might lead to differences in their susceptibility to uptake by phagocytes.

The second possibility is that there is some selectivity in the process of microbial uptake by phagocytes. Some studies have shown that sampling of luminal antigens can occur via M cell-mediated transcytosis, and that some proteins expressed on the apical surface of M cells can bind to particular microbial components and promote microbial uptake in a selective manner [30]. Other studies have shown that some phagocyte subsets sample luminal antigens by extending their dendrites between enterocytes or through M cells [6, 7], and that some receptors such as scavenger receptor A expressed on phagocytes can recognize particular bacterial components such as lipoteichoic acid and can induce phagocytosis [31]. In addition, molecules that recognize particular bacteria are likely involved in selective uptake. It has been reported that IgA can coat specific gut microbiota [32], and that IgA-coated microbiota can enter PP [33]. The selectivity by host cells such as M cells and phagocytes or molecules such as IgA might contribute to the above differences.

Second, what effects does this microbial uptake have on mucosal immunity? In this study, each phagocyte subset showed distinct immune properties (Fig 2); however, there was no significant correlation between the composition of gut microbiota engulfed by the various phagocyte subsets and their immune functions. This suggests that differences in cell types have a greater effect on immune function than microbial composition does. In contrast, *L*. *murinus* has been shown to induce IL-10 and TGF-β expression in colonic DCs and macrophages to promote regulatory T cell differentiation [34]. In this study, although all of the SILP cell subsets frequently engulfed *L*. *murinus*, we did not observe increased Tr1 cell induction in these cell subsets. In addition, a recent study demonstrated that the SILP CD64^+^CX3CR1^+^ macrophages, included in the CD11b^int^CD11c^int^ cell fraction of the SILP in this study, were essential for the generation of SFB-specific Th17 responses [35], indicating that microbial uptake by phagocytes affects host mucosal immunity. In this study, the low uptake of SFB by the SILP CD11b^int^CD11c^int^ cell fraction may have led to lower induction of Th17 cells by the cell subset. Moreover, CD11b^hi^ cells and CD11b^hi^CD11c^hi^ cells of PP, which engulfed SFB in this study, exhibited a potent ability to induce Th1, not Th17 cells, indicating that uptake of SFB by the PP phagocyte subsets is involved in the induction of Th1-cell differentiation. These results suggest that the ability to induce helper T cell subsets can vary with the composition of the microbial community. Further analyses of these phagocyte subsets under different microbial compositions, such as that of gnotobiotic mice, may reveal the relationship between the microbial uptake and immune function of each cell subset.

In conclusion, this study indicates that indigenous gut bacteria such as *L*. *murinus* and SFB are selectively taken up by small intestinal phagocytes that localize in the adjacent intestinal mucosa. Given that this was observed even in the steady state, we assume that such uptake is involved in maintaining mucosal homeostasis in the small intestine and establishing a symbiotic relationship between the gut microbiota and host. We believe that this study may provide new insights into the crucial interactions between phagocytes and gut microbiota during immune surveillance of the small intestinal mucosa, and that elucidation of the factors responsible for the selectivity of microbial uptake and effects of uptake on host mucosal immunity can lead to a better understanding of the mechanisms by which indigenous gut bacteria establish a symbiotic relationship with their host and help maintain mucosal homeostasis.

## Supporting Information

S1 Fig Composition of gut microbiota engulfed by phagocytes of specific subsets at the family level. Total DNA from each phagocyte subset and small intestinal content (SI content) was extracted and used for bacterial 16S rRNA gene amplicon sequencing. Bars indicate the microbial composition of each sample at the family level. Data represent the means of four independent experiments. Seven or eight C57BL/6 mice were pooled and used for PP or SILP experiments, respectively.
(TIF)
