# Supplementary figures and images for: Microbiota of the Small Intestine Is Selectively Engulfed by Phagocytes of the Lamina Propria and Peyer’s Patches
Source: PLoS One. 2016 Oct 4;11(10):e0163607. doi: 10.1371/journal.pone.0163607 (PMC5049916; doi:10.1371/journal.pone.0163607)

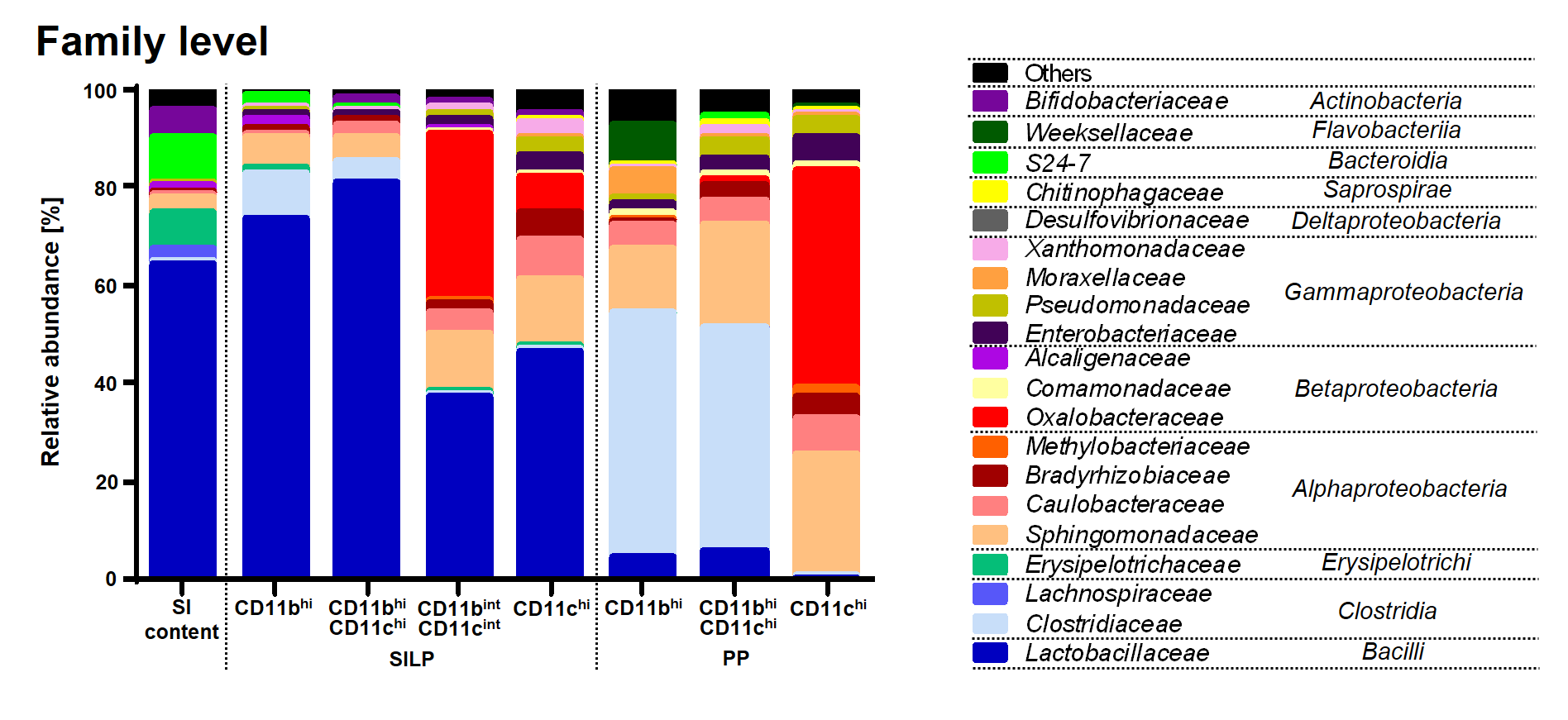

Supplement: S1 Fig — Total DNA from each phagocyte subset and small intestinal content (SI content) was extracted and used for bacterial 16S rRNA gene amplicon sequencing. Bars indicate the microbial composition of each sample at the family level. Data represent the means of four independent experiments. Seven or eight C57BL/6 mice were pooled and used for PP or SILP experiments, respectively. (TIF) [file pone.0163607.s001.tif]
